# Regulation of Adipogenesis and Thermogenesis through Mouse Olfactory Receptor 23 Stimulated by α-Cedrene in 3T3-L1 Cells

**DOI:** 10.3390/nu10111781

**Published:** 2018-11-16

**Authors:** Tao Tong, Jinju Park, Cheil Moon, Taesun Park

**Affiliations:** 1 Department of Food and Nutrition, Brain Korea 21 PLUS Project, Yonsei University, 50 Yonsei-ro, Seodaemun-gu, Seoul 03722, Korea; tongtao1028@163.com (T.T.); jeanzu@naver.com (J.P.); 2 Department of Brain and Cognitive Sciences, Daegu Gyeongbuk Institute of Science and Technology, Daegu 711-873, Korea; cmoon@dgist.ac.kr; 3 Convergence Research Advanced Centre for Olfaction, Daegu Gyeongbuk Institute of Science and Technology, Daegu 711-873, Korea

**Keywords:** olfactory receptors, MOR23, cAMP, α-cedrene, ectopic function, 3T3-L1 cells

## Abstract

Olfactory receptors (ORs) are G protein-coupled receptors that perform important physiological functions beyond their role as odorant detectors in the olfactory sensory neurons. In the present study, we describe a novel role for one of these ORs, mouse olfactory receptor 23 (MOR23), as a regulator of adipogenesis and thermogenesis in 3T3-L1 cells. Downregulation of MOR23 by small interfering RNA in 3T3-L1 cells enhanced intracellular lipid accumulation and reduced the oxygen consumption rate. In agreement with this phenotype, MOR23 deletion significantly decreased intracellular cyclic adenosine monophosphate (cAMP) levels and protein amounts of adenylyl cyclase 3 (ADCY3), protein kinase A catalytic subunit (PKA Cα), phospho-5′-adenosine monophosphate (AMP)-activated protein kinase (AMPK), and phospho-cAMP-responsive element-binding protein (CREB), along with upregulation of adipogenic genes and downregulation of genes involved in thermogenesis. Activation of MOR23 by α-cedrene, a novel natural ligand of MOR23, significantly reduced lipid content, increased the oxygen consumption rate, and stimulated reprogramming of the metabolic signature of 3T3-L1 cells, and these changes elicited by α-cedrene were absent in MOR23-deficient cells. These findings point to the role of MOR23 as a regulator of adipogenesis and thermogenesis in adipocytes.

## 1. Introduction

G protein-coupled receptors (GPCRs) constitute by far the largest receptor family in mammals and are involved in the regulation of virtually all cellular and physiological functions in the body. Owing to their ability to bind to ligands with high specificity and affinity, GPCRs are preferentially targeted for the development of new therapeutics and account for ~40% of the currently exploited drug targets [1]. Olfactory receptors (ORs) form the largest subfamily of GPCRs [2,3,4] and were originally postulated to be present in the olfactory epithelium exclusively. Nonetheless, further studies have shown that ORs are much more versatile than previously thought and are now emerging as general chemoreceptors that are found in various tissues, where they perform diverse patterns of regulatory functions. For example, ORs in the gut [5], spleen [6], liver [7], gastrointestinal tract [8], prostate [9], and testicles [10] appear to play specific signaling roles in the regulation of normal physiology and development. These observations make ORs promising markers and potential therapeutic targets in human diseases beyond the usefulness for the fragrance industry.

Using mode-of-action by network identification analysis, which is a validated means for the identification of targets and associated pathways of compounds [11,12,13,14], we have previously demonstrated that ORs are possible genetic mediators of high-fat diet–induced obesity progression in adipose tissues [15]. We have also reported that haploinsufficiency of adenylyl cyclase 3 (ADCY3), which is a downstream signal-transducing component of the olfactory signaling machinery, results in significantly increased visceral adiposity without hyperphagia in mice on either chow or high-fat diet [16]. At the same time, we found that α-cedrene, a sesquiterpene constituent of cedarwood oil, protects mice and rats from weight gain and metabolic aberrations without affecting their food intake, and these beneficial effects of α-cedrene are attenuated by ~50% in heterozygous ADCY3-null mice (unpublished finding). Subsequently, in an attempt to look for the specific OR isoform, we have tested the response of Hana3A cells (heterologously expressing ORs) to α-cedrene. The results have led to the identification of MOR23 as a molecular target of α-cedrene [7]. In human hepatocytes, a reduction in olfactory receptor 10J5 (OR10J5, human orthologue of MOR23) expression by specific small interfering RNA (siRNA) abrogates the lipid-lowering effect of α-cedrene [7]. In myoblasts, the knockdown of MOR23 inhibits the ability of α-cedrene to decrease the intramyocellular lipid accumulation induced by palmitic acid [17]. In the present study, we aimed to test whether MOR23 performs regulatory functions in adipogenesis and thermogenesis in murine adipocytes.

## 2. Materials and Methods

### 2.1. Reagents

Oil Red O were purchased from Sigma-Aldrich (Louis, MO, USA). α-Cedrene (batch No. KDCB212DA01, purity: 99.6%) was supplied by Kwang Dong Pharmaceutical Co. (Seoul, South Korea). The antibodies against glyceraldehyde-3-phosphate dehydrogenase (GAPDH) (#2118; 1:5000 dilution), 5′-adenosine monophosphate (AMP)-activated protein kinase (AMPK, #2532, 1:1000 dilution), cyclic adenosine monophosphate (cAMP)-responsive element-binding protein (CREB, #9197, 1:1000 dilution), phospho-AMPK (#2531; 1:1000 dilution), phospho-CREB (#9198; 1:1000 dilution), and protein kinase A catalytic subunit (PKA Cα, cat. #4782, 1:1000 dilution) were purchased from Cell Signaling Technology (Danvers, MA, USA). An antibody against ADCY3 (sc-588; 1:200 dilution) was acquired from Santa Cruz Biotechnology (Dallas, TX, USA). A horseradish peroxidase (HRP)-conjugated anti-rabbit IgG antibody (1:5000 dilution; Santa Cruz Biotechnology cat. # sc-2004; secondary antibody) was used for immunoblotting procedures.

### 2.2. Cell Culture

3T3-L1 murine fibroblasts acquired from American Type Culture Collection (Manassas, VA, USA) were cultured in Dulbecco’s modified Eagle’s medium containing streptomycin (50 mg/mL), penicillin (50 U/mL) (Life Technologies, Carlsbad, CA, USA), and 10% of fetal bovine serum (HyClone, Logan, UT, USA) in a humidified atmosphere containing 5% of CO_2_ at 37 °C. To promote the differentiation of 3T3-L1 cells into adipocytes, we treated confluent cultures with and insulin (10 μg/mL, Sigma-Aldrich), 3-isobutyl-1-methylxanthine (0.5 mM, Sigma-Aldrich), and dexamethasone (0.25 mM, Sigma-Aldrich) (simultaneously). After 2 days, dexamethasone and 3-isobutyl-1-methylxanthine were removed, and incubation with insulin was continued for another 2 days. The growth medium was refreshed at 2-day intervals until complete adipocytic differentiation.

### 2.3. cAMP Assay

cAMP was extracted from 3T3-L1 cells using HCl (0.1 M, Enzo Life Sciences, Montgomery County, PA, USA). After that, the intracellular concentration of cAMP in the lysates was determined according to methods provided by cAMP ELISA kit (Enzo Life Sciences). Competitive binding is the principle of the cAMP assay. cAMP within the sample competes with a fixed amount of horseradish peroxidase–labeled cAMP for sites on the monoclonal antibody. cAMP was quantified by measurement of optical density (OD) at 450 nm on a microplate reader (Versa Max, Molecular Devices, San Jose, CA, USA).

### 2.4. siRNA Knockdown

3T3-L1 preadipocytes were seeded in 12-well plates (10^5^ cells per well) and transfected with a pool of nontargeting siRNA control oligonucleotides (ON-TARGET plus Control pool, D-001810-10-05; Dharmacon), or siRNA oligonucleotides against mouse MOR23 (80 nM, ON-TARGET plus smart pool, L-022371-02; Dharmacon, Lafayette, CO, USA) using Lipofectamine 2000 (Life Technologies, Carlsbad, CA, USA). Target sequences for siRNA against mouse MOR23 were as follows: CAAUGGGUUAUGAUCGUUA, CUGAAGUGAUAGAGUUCGU, GGUGUAAGUUCAUUUGUAA, and CCAUAGGGCUGAUAUUUAU. Forty-eight hours after the transfection, the cells were stimulated for adipogenesis. The MOR23 ON-TARGET plus smart pool was a mixture of four siRNAs. The knockdown efficiency of siRNA was assessed by semiquantitative RT-PCR.

### 2.5. Oil Red O Staining

The cells were washed with warm phosphate-buffered saline (Sigma-Aldrich), fixed with neutral formaldehyde (10%, Sigma-Aldrich) at room temperature for 2–3 h, then rinsed quickly with isopropanol (60%, Sigma-Aldrich) and allowed to dry. After that, we used Oil Red O (Sigma-Aldrich; 0.5% *w*/*v* diluted 3:2 with double-distilled water) to stain the cells and incubated the cells at room temperature for 2–3 h prior to 3–4 washes with distilled water. Images were then acquired by means of the Olympus microscope IX71 (Olympus, Center Valley, PA, USA). To release Oil Red O from steatosis staining, 500 μL of isopropanol was added into each well prior to incubation for 10 min at room temperature. After being transferred to a 96-well plate, the optical density of the isopropanol solution at a wavelength of 490 nm was determined by means of a microplate reader (Versa Max).

### 2.6. Measurement of the Oxygen Consumption Rate

Mitochondrial function was quantified using the Seahorse XF-24 analyzer (Seahorse Bioscience, Billerica, MA, USA). Measurements were carried out on day 12 after the induction of differentiation. The sensor cartridge was hydrated with Seahorse XF Calibrant (Seahorse Bioscience) overnight at 37 °C in a non-CO_2_ incubator. The assay medium was prepared in Seahorse XF Base Medium (Seahorse Bioscience) supplemented with sodium pyruvate (110 mg/L, Sigma-Aldrich), l-Glutamax (4 mM, Sigma-Aldrich), and d-glucose (4500 mg/L, Sigma-Aldrich) and were adjusted to pH 7.4 using NaOH (0.1 M, Sigma-Aldrich). Cells were incubated at 37 °C in a non-CO_2_ incubator in assay medium for 1 h prior to measurement. Changes in the oxygen consumption rate were measured over time in response to the synchronous addition of oligomycin (1 μM), fluoro-carbonyl cyanide phenylhydrazone (1 μM, FCCP), and rotenone/antimycin A (0.5 μM, XF Cell Mito Stress Kit, Seahorse Bioscience) at specific time points. H^+^ leakage was calculated as the difference in the oxygen consumption rate between the cells after the addition of oligomycin and the cells after the addition of rotenone/antimycin. Maximal mitochondrial oxygen consumption was computed as the difference in the oxygen consumption rate between the cells after the addition of FCCP and the cells after the addition of rotenone/antimycin. All the measurements were normalized to total protein content. The cells were harvested in lysis buffer and incubated at −20 °C for 20 min. After that, the lysates were centrifuged for 20 min at 13,000× *g* at 4 °C. The protein concentrations were determined according to the Bradford method (Bio-Rad, Hercules, CA, USA).

### 2.7. RNA Extraction and PCR

Total RNA was extracted form 3T3-L1 cells using the TRIzol Reagent (Life Technologies). cDNA synthesis was performed with RNase inhibitor (40 U/μL, Invitrogen, Carlsbad, CA, USA), reverse transcriptase (200 U/μL, Invitrogen), dithiothreitol (0.1 M, Invitrogen), dNTP (2.5 mM, Invitrogen), 5× RT buffer diluted to 1× (Invitrogen), and total RNA (1 μg) in a total reaction volume of 40 μL at 37 °C for 2 h. For semi-quantitative PCR, the amounts of mRNA were measured by means of the 5× PCR Master Mix (Intron, Seoul, Korea) with GAPDH as an internal control. Quantitative PCR was next carried out using the CFX Connect™ Real-Time PCR Detection System (Bio-Rad, Hercules, CA, USA) and iQ SYBR green supermix (Bio-Rad). Primer sequences are presented in Table 1. The gene expression data were normalized to GAPDH. The results on the optical density ratio of a target gene to GAPDH are presented as mean ± standard error of mean (SEM) of at least three independent experiments.

### 2.8. Protein Extraction and Western Blotting Assay

3T3-L1 cells were harvested in lysis buffer, which containing leupeptin (1 μg/mL, Sigma-Aldrich), pepstatin A (1 μg/mL, Sigma-Aldrich), Triton X-100 (1%, Sigma-Aldrich), Tris-HCl (100 mM, pH 7.4, Sigma-Aldrich), aprotinin (2 μg/mL, Sigma-Aldrich), phenylmethylsulfonyl fluoride (1 mM, Sigma-Aldrich), NaCl (50 mM, Sigma-Aldrich), sodium pyrophosphate (50 mM, Sigma-Aldrich), NaF (50 mM, Sigma-Aldrich), ethylenediaminetetraacetic acid (EDTA, 5 mM, Sigma-Aldrich), and orthovanadate (100 mM, Sigma-Aldrich). After that, the protein samples were centrifuged for 20 min at 13,000× *g* at 4 °C. The protein concentrations were determined according to the Bradford method (Bio-Rad).

For Western blotting analysis, protein was separated by 10% sodium dodecyl sulfate (Sigma-Aldrich) polyacrylamide (Bio-Rad) gel electrophoresis prior to electrophoretically transferring to nitrocellulose membranes (Amersham Biosciences, Piscataway, NJ, USA). The membranes were blocked with bovine serum albumin (5%, Sigma-Aldrich), and then incubated with primary antibodies overnight at 4 °C prior to incubation with the corresponding secondary antibodies. We used the ECL Chemiluminescent Detection Reagent (GE Healthcare, Buckinghamshire, UK) to detect and visualize the protein bands. Images were captured with a LuminoGraph system (WSE-6100, ATTO, Tokyo, Japan). The results on the optical density ratio of target proteins to either GAPDH or tubulin are presented as mean ± SEM of at least three independent experiments.

### 2.9. Statistical Analysis

Student’s *t*-test was carried out to determine significance of the differences between the two groups. All statistical analyses were conducted using the GraphPad Prism 7 software (GraphPad, San Diego, CA, USA), and significance was set at * *p* < 0.05, ** *p* < 0.01 and *** *p* < 0.001.

## 3. Results

### 3.1. A Reduction in MOR23 Expression by Specific siRNA Increases Intracellular Lipid Accumulation

**Figure 1:**
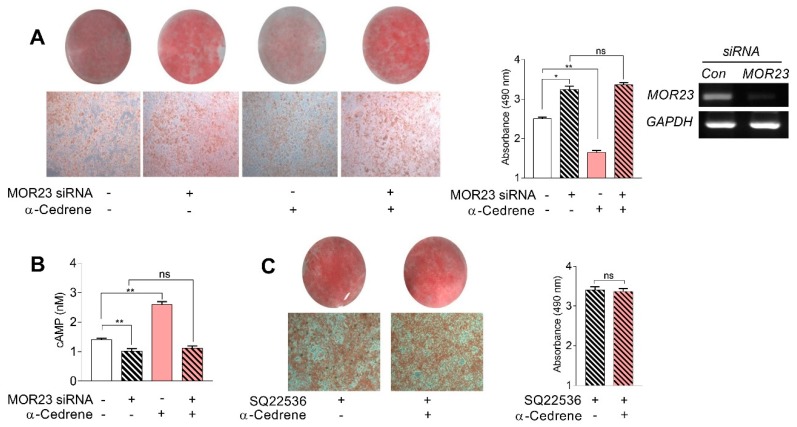
A reduction in mouse olfactory receptor 23 (MOR23) expression by specific siRNA increases intracellular lipid accumulation and abrogates the lipid-lowering effect of α-cedrene in 3T3-L1 cells. 3T3-L1 preadipocytes were transfected with MOR23 siRNA, stimulated to differentiate in culture 2 days after transfection in the presence of 100 μM α-cedrene or 50:50 (*v*/*v*) dimethyl sulfoxide (DMSO)/acetonitrile (vehicle). Oil Red O staining and cAMP assay were performed after adipocyte induction. (**A**) Oil red O staining of 3T3-L1 cells transfected with MOR23 siRNA with or without 100 μM α-cedrene. Representative photomicrographs (×100) are shown in the left panel. Right panel shows spectrophotometric quantification of Oil Red O staining. The knockdown efficiency by siRNA was monitored by semi-quantitative reverse transcription-polymerase chain reaction (RT-PCR). (**B**) Cyclic adenosine monophosphate (cAMP) level of 3T3-L1 cells transfected with MOR23 siRNA with or without 100 μM α-cedrene. (**C**) Oil red O staining of 3T3-L1 cells exposed to adenylyl cyclase (ADCY) inhibitor for 24 h with or without 100 μM α-cedrene. Representative photomicrographs (×100) are shown in the left panel. Right panel shows spectrophotometric quantification of Oil Red O staining. The full-length gels are presented in Supplementary Figure S1. The values represent the means ± SEM, *n* = 3. Significant differences between groups are indicated by asterisks; * *p* < 0.05; ** *p* < 0.01; ns, not significant (*p* > 0.05).

To test whether MOR23-mediated signaling pathways are involved in adipogenesis, we knocked down MOR23 in 3T3-L1 cells, using MOR23-specific siRNA (a pool of four MOR23-specific oligonucleotides; Figure 1A). The MOR23 knockdown significantly increased intracellular lipid accumulation in 3T3-L1 cells, as determined by confocal microscopy after staining with Oil Red O (Figure 1A). These findings were confirmed by colorimetric quantification of the intracellular triglyceride concentration in 3T3-L1 cells transfected with MOR23 siRNA (Figure 1A). The MOR23 ligand, α-cedrene, significantly deceased the intracellular lipid accumulation in 3T3-L1 cells (Figure 1A). Nevertheless, we found that the beneficial changes in lipid accumulation elicited by α-cedrene were completely blocked by the siRNA-mediated knockdown of MOR23 (Figure 1A). The MOR23 knockdown also significantly decreased intracellular cAMP levels in 3T3-L1 cells (Figure 1B). Treatment of 3T3-L1 cells with α-cedrene increased the intracellular cAMP levels, and this change elicited by α-cedrene was abrogated by the siRNA-mediated knockdown of MOR23 (Figure 1B). Next, we evaluated the effect of α-cedrene on the regulation of lipid accumulation in the presence of SQ22536 (a commercially available inhibitor of adenylyl cyclases; ADCYs), because ADCY3 is a pivotal downstream molecule of the OR-mediated signaling pathways [18]. Similarly, we found that effects of α-cedrene on the regulation of lipid accumulation disappeared in the presence of SQ22536 (Figure 1C).

### 3.2. A Reduction in MOR23 Expression Impaired the cAMP Signaling Pathway and Upregulated the Expression of Adipogenic Genes

**Figure 2:**
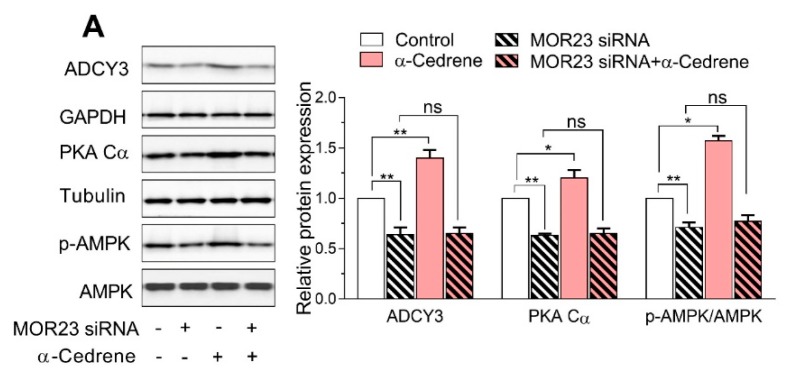

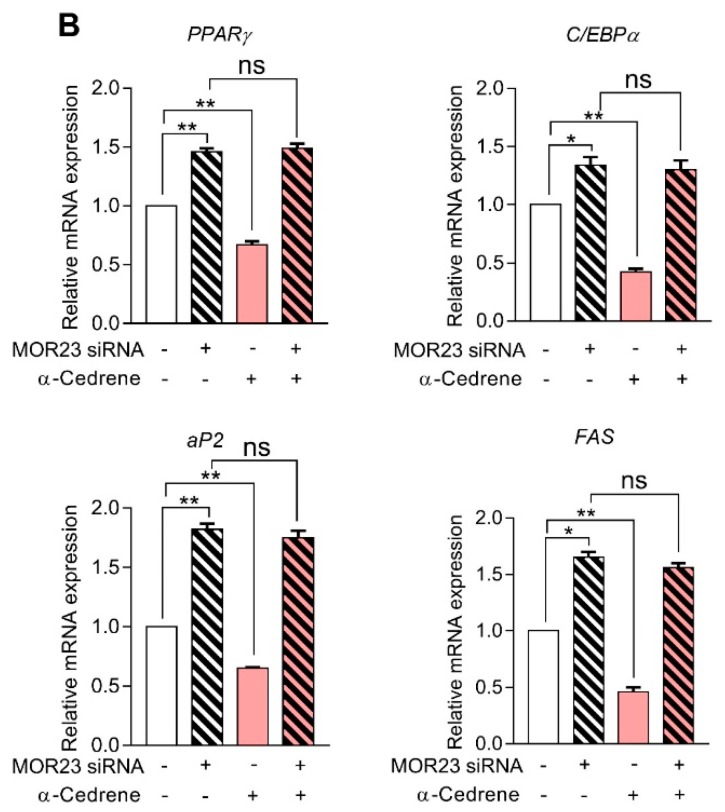
A reduction in MOR23 expression impairs the cAMP signaling pathway and upregulated the mRNA expression of adipogenic genes. 3T3-L1 preadipocytes were transfected with MOR23 siRNA, stimulated to differentiate in culture 2 days after transfection in the presence of 100 μM α-cedrene or 50:50 (*v*/*v*) DMSO/acetonitrile (vehicle). Protein and RNA extraction were performed after adipocyte induction. (**A**) Western blot analysis of adenylyl cyclase 3 (ADCY3), PKA Cα, and phosphor-AMPK in 3T3-L1 cells transfected with MOR23 siRNA with or without 100 μM α-cedrene. (**B**) RT-PCR analysis of the mRNA expression of Peroxisome proliferator-activated receptor γ (*PPARγ*), *C/EBP*α, *aP2*, and *FAS* in 3T3-L1 cells transfected with MOR23 siRNA with or without 100 μM α-cedrene. The full-length blots are presented in Supplementary Figure S2. The values represent the means ± SEM, *n* = 3. Significant differences between groups are indicated by asterisks; * *p* < 0.05; ** *p* < 0.01; ns, not significant (*p* > 0.05).

The MOR23 knockdown significantly impaired the cAMP signaling pathway in 3T3-L1 cells, judging by decreased protein levels of ADCY3, PKA Cα, and phospho-AMPK (Figure 2A). Moreover, a reduction in MOR23 expression upregulated mRNA expression of adipogenic genes, such as *PPARγ*, *C/EBPα*, *aP2*, and *FAS* (Figure 2B). α-Cedrene significantly enhanced the cAMP signaling pathway in 3T3-L1 cells, as demonstrated by increased protein levels of ADCY3, PKA, and phospho-AMPK and decreased mRNA expression of adipogenic genes (*PPARγ*, *C/EBPα*, *aP2*, and *FAS*) (Figure 2A,B). These α-cedrene–induced changes in protein and mRNA expression profiles were markedly attenuated in MOR23-depleted cells (Figure 2A,B).

### 3.3. MOR23 Depletion Decreases Thermogenesis 3T3-L1 cells

**Figure 3:**
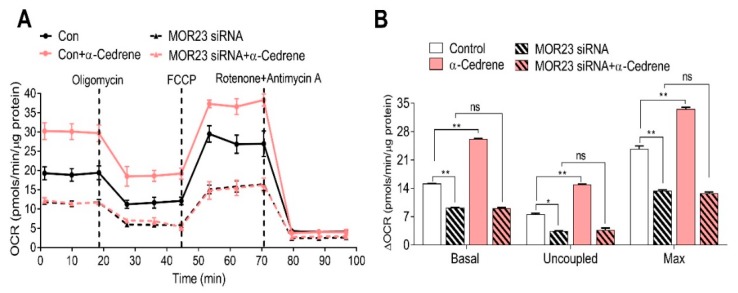
MOR23 depletion decreases thermogenesis and abolishes the thermogenic effect of α-cedrene in 3T3-L1 cells. 3T3-L1 preadipocytes were transfected with MOR23 siRNA, stimulated to differentiate in culture 2 days after transfection in the presence of 100 μM α-cedrene or 50:50 (*v*/*v*) DMSO/acetonitrile (vehicle). Oxygen consumption rate measurement was performed after adipocyte induction using the Seahorse XF-24 analyzer. (**A**) Oxygen consumption rate were measured in differentiated 3T3-L1 adipocytes in basal conditions, or in response to sequential treatment with 1 μM oligomycin (to block ATP synthesis), 1 μM FCCP (respiratory chain uncoupler), and 0.5 μM rotenone/antimycin A (inhibitor of respiratory chain complex I and complex III, respectively). (**B**) ΔOCR is calculated by subtracting oxygen consumption rate (OCR) measured after rotenone/antimycin A addition from basal OCR, from OCR after oligomycin addition, or from OCR after FCCP addition. All data are mean ± SEM, *n* = 3. Significant differences between groups are indicated by asterisks; * *p* < 0.05; ** *p* < 0.01; ns, not significant (*p* > 0.05).

To determine whether MOR23 directly alters thermogenesis in adipocytes, we evaluated the oxygen consumption rate (OCR) as a measure of oxidative phosphorylation in 3T3-L1 cells using the Seahorse XF-24 analyzer. The MOR23 depletion significantly decreased basal respiration, H^+^ leakage, and maximal respiration capacity (Figure 3A,B). α-Cedrene treatment significantly increased basal respiration, H^+^ leakage, and maximal respiration capacity of 3T3-L1 cells (Figure 3A,B). The abilities of α-cedrene to induce oxygen consumption were strongly attenuated in MOR23-depleted cells (Figure 3A,B), indicating that α-cedrene affects energy expenditure in a MOR23-dependent manner.

### 3.4. MOR23 Depletion Decreases the Expression of Thermogenic and Mitochondrial Genes

**Figure 4:**
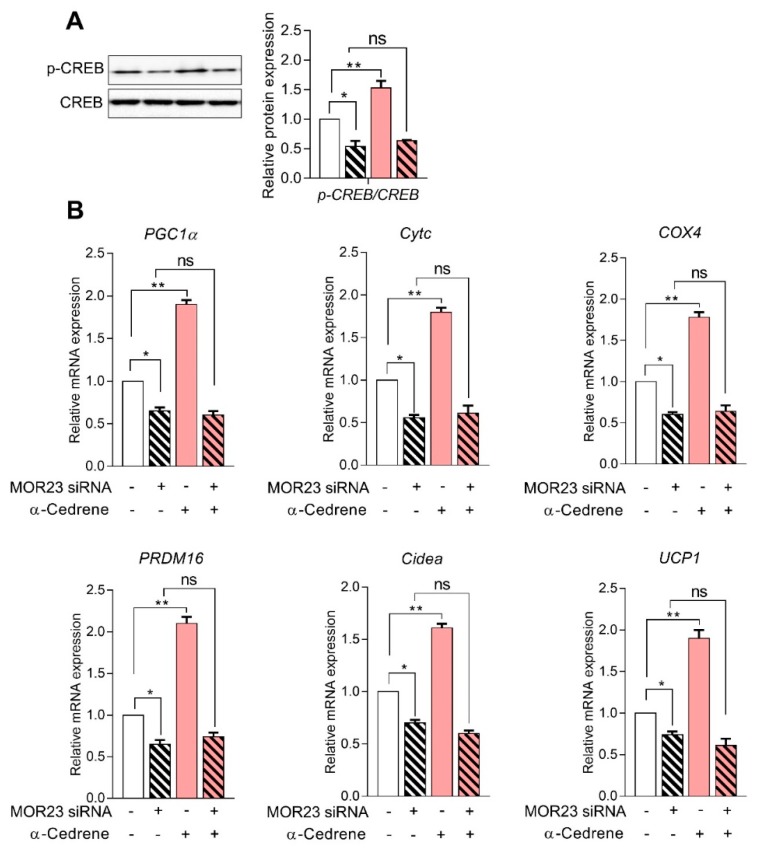
MOR23 depletion decreases the expression of thermogenic and mitochondrial genes. 3T3-L1 preadipocytes were transfected with MOR23 siRNA, stimulated to differentiate in culture 2 days after transfection in the presence of 100 μM α-cedrene or 50:50 (*v*/*v*) DMSO/acetonitrile (vehicle). Protein and RNA extraction were performed after adipocyte induction. (**A**) Western blot analysis of phosphor-CREB in 3T3-L1 cells transfected with MOR23 siRNA with or without 100 μM α-cedrene. (**B**) RT-PCR analysis of the mRNA levels of *peroxisome proliferator-activated receptor gamma coactivator 1-alpha* (*PGC-1α*), cytochrome c (*Cytc*), *cytochrome c oxidase subunit 4* (*COX4*), *PR domain containing 16* (*PRDM16*), *cell death activator CIDE-A* (*Cidea*), and *uncoupling protein 1* (*UCP1*) in 3T3-L1 cells transfected with MOR23 siRNA with or without 100 μM α-cedrene. The full-length blots are presented in Supplementary Figure S3. The values represent the means ± SEM, *n* = 3. Significant differences between groups are indicated by asterisks; * *p* < 0.05; ** *p* < 0.01; ns, not significant (*p* > 0.05).

It has been established that phosphorylation of CREB by the cAMP–PKA pathway activates the expression of peroxisome proliferator-activated receptor gamma coactivator 1-alpha (*PGC-1α*), which induces the transcription of downstream thermogenic genes, such as uncoupling protein 1 (*UCP1*), PR domain containing 16 (*PRDM16*), and cell death activator CIDE-A (*Cidea*), in adipocytes [19,20]. Consistent with the OCR data, the knockdown of MOR23 significantly decreased the amounts of phospho-CREB and reduced mRNA expression of thermogenic (*PGC-1α*, *PRDM16*, *Cidea*, and *UCP1*) and mitochondrial genes, such as cytochrome c (*Cytc*) and cytochrome c oxidase subunit 4 (*COX4*). (Figure 4A,B). In line with these physiological responses, α-cedrene increased phosphorylation of CREB and upregulated thermogenic (*PGC-1α*, *PRDM16*, *Cidea*, and *UCP1*) and mitochondrial (*Cytc* and *COX4*) gene expression programs in 3T3-L1 cells (Figure 4A,B). The abilities of α-cedrene to induce thermogenic and mitochondrial gene expression programs were strongly attenuated in MOR23-depleted cells (Figure 4A,B).

## 4. Discussion

In olfactory sensory neurons of the olfactory epithelium, a cAMP-dependent pathway mediates canonical OR signaling. In this pathway, ORs are coupled to Gαolf, a protein homologous to Gαs, resulting in activation of an adenylyl cyclase and production of cAMP [21,22]. Furthermore, it was recently reported that in some nonchemosensory cells, such as sperm [23], keratinocytes [24], and myoblasts [25], activation of ectopically expressed ORs leads to canonical OR signaling events as well. In the present study, we found that MOR23 deletion in murine 3T3-L1 preadipocytes significantly decreased intracellular cAMP levels. Moreover, stimulation of 3T3-L1 cells with the MOR23 ligand α-cedrene significantly increased the intracellular cAMP concentration. By gene silencing and by means of a specific inhibitor of a key enzyme typically activated by a GPCR, we demonstrated that the elevation of cAMP levels after α-cedrene treatment is caused by activation of MOR23. Our results suggest that 3T3-L1 cells have a canonical OR signaling pathway.

**Figure 5:**
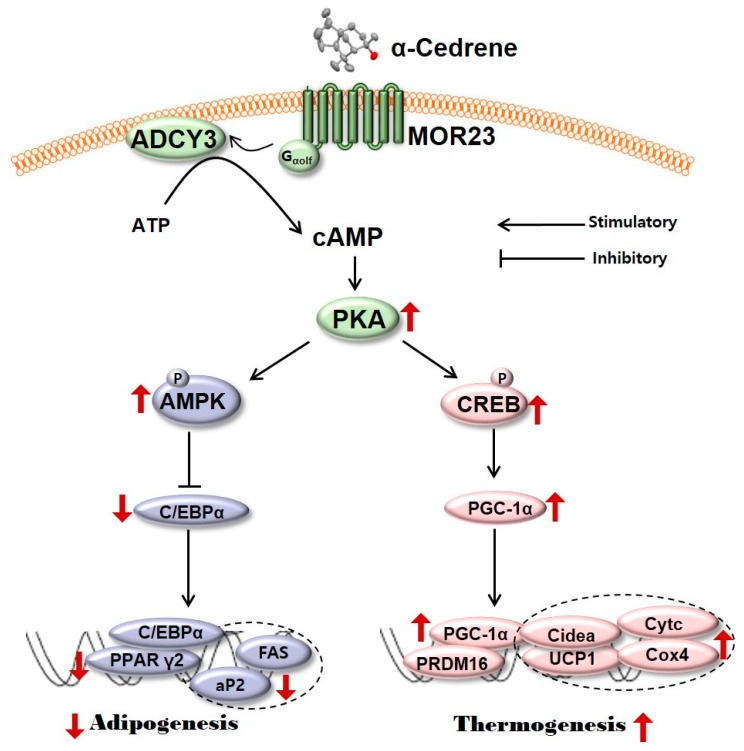
A schematic diagram illustrating the proposed mechanism by which MOR23 responding to α-cedrene regulates adipogenesis and thermogenesis. MOR23, mouse olfactory receptor 23; ADCY3, adenylyl cyclase 3; cAMP, cyclic adenosine monophosphate; PKA, protein kinase A; AMPK, 5′-adenosine monophosphate (AMP)-activated protein kinase; CREB, cAMP-responsive element-binding protein; C/EBPα, CCAAT/enhancer binding-protein α; PGC-1α, peroxisome proliferator-activated receptor gamma coactivator 1-alpha; PPARγ2, peroxisome proliferator-activated receptor γ2; aP2, adipocyte fatty acid binding protein; FAS, fatty acid synthase; PRDM16, PR domain containing 16; Cidea, cell death activator CIDE-A; UCP1, uncoupling protein 1; Cytc, cytochrome c; Cox4, cytochrome c oxidase subunit 4.

Adipogenesis is the differentiation of fibroblast like preadipocytes into mature lipid laden adipocytes [26]. In the present study, in order to test whether MOR23 has a potential role in adipogenesis, MOR23 was silenced in 3T3-L1 preadipocytes two days before differentiation. Silencing of MOR23 significantly promoted differentiation as demonstrated by substantially increased accumulation of neutral lipids measured by Oil Red O staining (Figure 1A). It is widely accepted that cAMP signaling pathways are pivotal for the regulation of adipocyte development and function [27,28,29]. For example, the elevation of intracellular cAMP levels by treatment with an ADCY activator, forskolin, suppresses adipogenesis as confirmed by both the morphological phenotype (Oil Red O staining of the lipid drops) and the mRNA expression of key adipogenic transcription factors in 3T3-L1 preadipocytes [30]. cAMP acts mainly through its binding to PKA, which are ubiquitous intracellular cAMP effectors that regulate multiple processes [31,32]. It is known that binding of cAMP to the regulatory subunit of PKA unleashes the catalytic subunit so that it can phosphorylate its protein substrates (that affect lipid metabolism), for instance, AMPK [33,34] and CREB [19,20] in adipocytes of all colors and origins. Phospho-AMPK inhibits differentiation of preadipocytes by downregulating PPARγ and C/EBPα, which are the central regulators of adipogenesis and lipid storage in adipocytes [35,36,37,38]. In parallel, phospho-CREB activates the expression of PGC-1α, which induces the transcription of downstream thermogenic genes, including UCP1, PRDM16, and Cidea [20,39]. In the present study, a loss of MOR23 significantly decreased the protein levels of ADCY3, PKA Cα, phospho-AMPK, and phospho-CREB in 3T3-L1 cells (Figure 2 and Figure 4). These results indicate that in 3T3-L1 cells, MOR23 may be associated with the regulation of cAMP-and-PKA–mediated signaling pathways involved in thermogenesis and adipogenesis (Figure 5).

ORs constitute nearly 50% of the ~800 GPCRs in humans, yet ~90% of ORs remain orphan receptors (unknown ligands) [40]. One study on a number of ORs that have been functionally matched with their cognate ligands revealed that mammalian ORs vary along a continuum of tuning breadth [41]. That is, some receptors are broadly tuned, responding to a large number of odorants that occupy a large area of odorant space, whereas others are narrowly tuned, i.e., highly specific for only a small number of odorants [41]. In a cell-based assay for odorant-induced changes in intracellular cAMP, we observed that aside from α-cedrene, neither MOR23 nor OR10J5 responded to the odorant phytochemicals that were found to attenuate lipid accumulation in 3T3-L1 cells (data not shown). Thus, we can hypothesize that MOR23 is a narrowly tuned receptor, i.e., responding only to a small number of odorants.

A specific OR is known to be functionally expressed in a wide range of tissues and cell types where it performs diverse functions [18]. It is known that MOR23 is functionally expressed in mouse spermatogenic cells and sperm, and its activation increases intracellular Ca^2+^ levels and regulates sperm motility [10]. Moreover, Griffin et al. demonstrated that a knockdown of MOR23 via siRNA in myoblasts significantly inhibits their migration, cell–cell adhesion, and formation of multinucleated myotubes [25]. OR10J5, the human ortholog of MOR23, has been demonstrated to be a key regulator of angiogenesis and to stimulate migration of human umbilical vein endothelial cells by activating the Ca^2+^-dependent AKT signal transduction pathway [42]. Recently, we found that OR10J5 is deeply involved in the regulation of lipid accumulation in human hepatocytes: the siRNA-mediated knockdown of OR10J5 increases intracellular lipid accumulation along with upregulation of lipogenic genes and downregulation of genes related to fatty acid oxidation [7]. Therefore, it is intriguing to consider the possibility that ectopically expressed MOR23 may serve as a sensitive and selective chemoreceptor that influences many physiological processes in nonolfactory tissues.

Previous studies of MOR23 function in vivo revealed that in gastrocnemius muscles of mice, electroporation of a plasmid expressing MOR23 siRNA blunts barium chloride-induced muscle regeneration and leaves many branched, unfused myofibers, commonly associated with muscle dystrophy [25]. In addition, stimulation of cAMP production in muscle can attenuate degeneration or promote regeneration in rodent models of necrotic muscle injury [43] and Duchenne’s muscular dystrophy [44,45,46]. cAMP also has a role in controlling adipocyte development and function through regulating the expressions of genes related to adipogenesis and thermogenesis. Hence, it is reasonable to speculate that the production of variable cellular effects via the MOR23 signaling in different cell types or tissues could be explained by the ability of cAMP to produce different effects depending on the cell type or tissues.

## 5. Conclusions

We showed here that MOR23 plays a key role in the regulation of adipogenesis and thermogenesis in 3T3-L1 cells: downregulation of MOR23 by siRNA increased intracellular lipid accumulation and reduced the oxygen consumption rate; MOR23 activation by its natural ligand α-cedrene significantly increased the oxygen consumption rate and reduced triglyceride accumulation, and these changes elicited by α-cedrene were abrogated by the siRNA-mediated knockdown of MOR23. In summary, our findings indicate that in addition to its participation in olfaction, ectopically expressed MOR23 can be considered a chemosensor playing a critical role in energy and lipid metabolism of 3T3-L1 adipocytes. Many unanswered questions relating to how MOR23 precisely regulates the development of obesity in vivo would warrant subsequent studies in mice lacking MOR23 in a tissue-specific manner.

## Figures and Tables

**Table 1:** Primer sequences.

Type	Gene Description	Sequences (5′→3′)
Mouse	Mouse olfactory receptor 23 (*MOR23*)	F: CAAGGCACACATTCCCTTGC
		R: TTCCCATATCCTTGGCAGGC
	Peroxisome proliferator-activated receptor γ2 (*PPARγ2*)	F: TTCGGAATCAGCTCTGTGGA
		R: CCATTGGGTCAGCTCTTGTG
	CCAAT/enhancer binding-protein α (*C/EBPα*)	F: TCAGCTTACAACAGGCCAGG
		R: ACACAAGGCTAATGGTCCCC
	Adipocyte fatty acid binding protein (*aP2*)	F: CATGCGACAAAGGCAGAAAT
		R: GTTACAAGGCAAGGAAGGGC
	Fatty acid synthase (*FAS*)	F: CAGCCAGGAGAATCGCAGTA
		R: CTGCGATGAAGAGCATGGTT
	Peroxisome proliferator-activated receptor gamma coactivator 1-alpha (*PGC-1α*)	F: TAAATCTGCGGGATGATGGA
		R: GTTTCGTTCGACCTGCGTAA
	Uncoupling protein 1 (*UCP1*)	F: GGTTTGCACCACACTCCTG
		R: ACATGGACATCGCACAGCTT
	PR domain containing 16 (*PRDM16*)	F: GGACCTTTTTGACAGCAGCA
		R: GGGGGCAAAGCATTTAACTC
	Cytochrome c (*Cytc*)	F: ACACTGTGGAAAAGGGAGGC
		R: GCACTGGTTAACCCAAGCAA
	Cytochrome c oxidase subunit 4 (*COX4*)	F: GGAAAACGTCTGCCGGAAA
		R: AAGCATCGCGGGAATCAGG
	Cell death activator CIDE-A (*Cidea*)	F: GGAATCTGCTGAGGTTTATG
		R: ATCCCACAGCCTATAACAGA
	Glyceraldehyde-3-phosphate dehydrogenase (*GAPDH*)	F: GTGATGGCATGGACTGTGGT
		R: GGAGCCAAAAGGGTCATCAT

## Supplementary Materials

The following are available online at http://www.mdpi.com/2072-6643/10/11/1781/s1, Figure S1: Full length agarose gels relative to Figure 1A. Yellow boxes indicate the cropping lines used to generate the figures, Figure S2: Full length western blot membranes relative to Figure 2A. Red boxes indicate the cropping lines used to generate the figures, Figure S3: Full length western blot membranes relative to Figure 4A. Red boxes indicate the cropping lines used to generate the figures.
