# Supplementary material for: Regulation of Adipogenesis and Thermogenesis through Mouse Olfactory Receptor 23 Stimulated by α-Cedrene in 3T3-L1 Cells
Source: Nutrients. 2018 Nov 16;10(11):1781. doi: 10.3390/nu10111781 (PMC6265911; doi:10.3390/nu10111781)
Supplement: Supplementary file 1 [file nutrients-10-01781-s001.pdf]

Supplementary Information

Regulation of adipogenesis and thermogenesis through mouse olfactory receptor 23 stimulated by α-cedrene in 3T3-L1 cells

Tao Tong [1], Jinju Park [1], Cheil Moon[2,3], and Taesun Park [1,*]

[1] Department of Food and Nutrition, Brain Korea 21 PLUS Project, Yonsei University, 50 Yonsei-ro, Seodaemun-gu, Seoul 03722, South Korea; tongtao1028@163.com (T.T.); jeanzu@naver.com (J.P.); tspark@yonsei.ac.kr (T.P.)

[2] Department of Brain and Cognitive Sciences, Daegu Gyeongbuk Institute of Science and Technology, Daegu, 711-873, South Korea; cmoon@dgist.ac.kr (C.M.)

[3] Convergence Research Advanced Centre for Olfaction, Daegu Gyeongbuk Institute of Science and Technology, Daegu, 711-873, South Korea; cmoon@dgist.ac.kr (C.M.)

Correspondence: tspark@yonsei.ac.kr; Tel.: +82-2-2123-3123; Fax: +82-2-365-3118

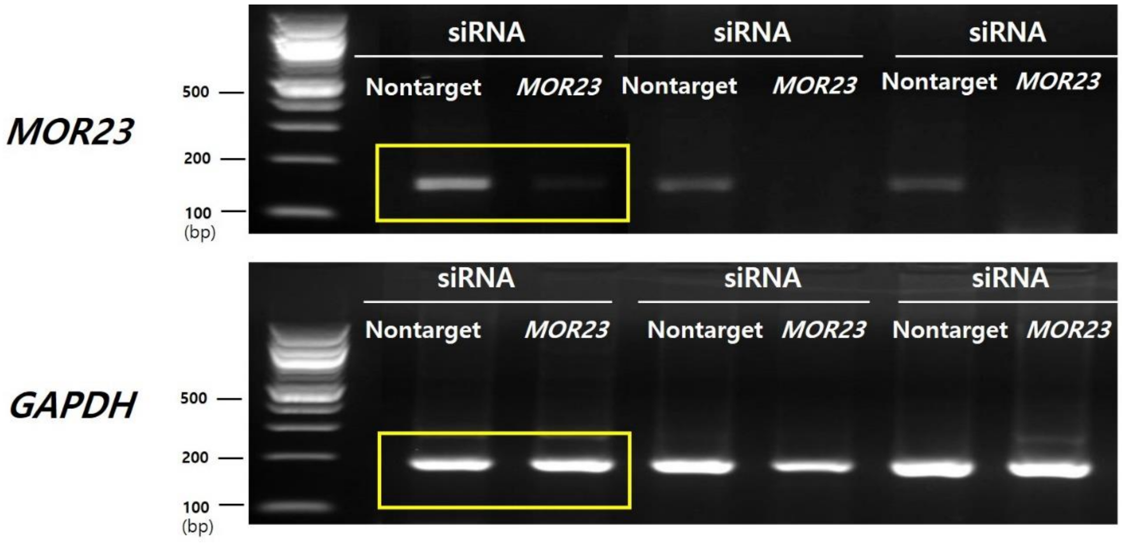

Supplementary Figure 1. Full length agarose gels relative to Figure 1A. Yellow boxes indicate the cropping lines used to generate the figures.

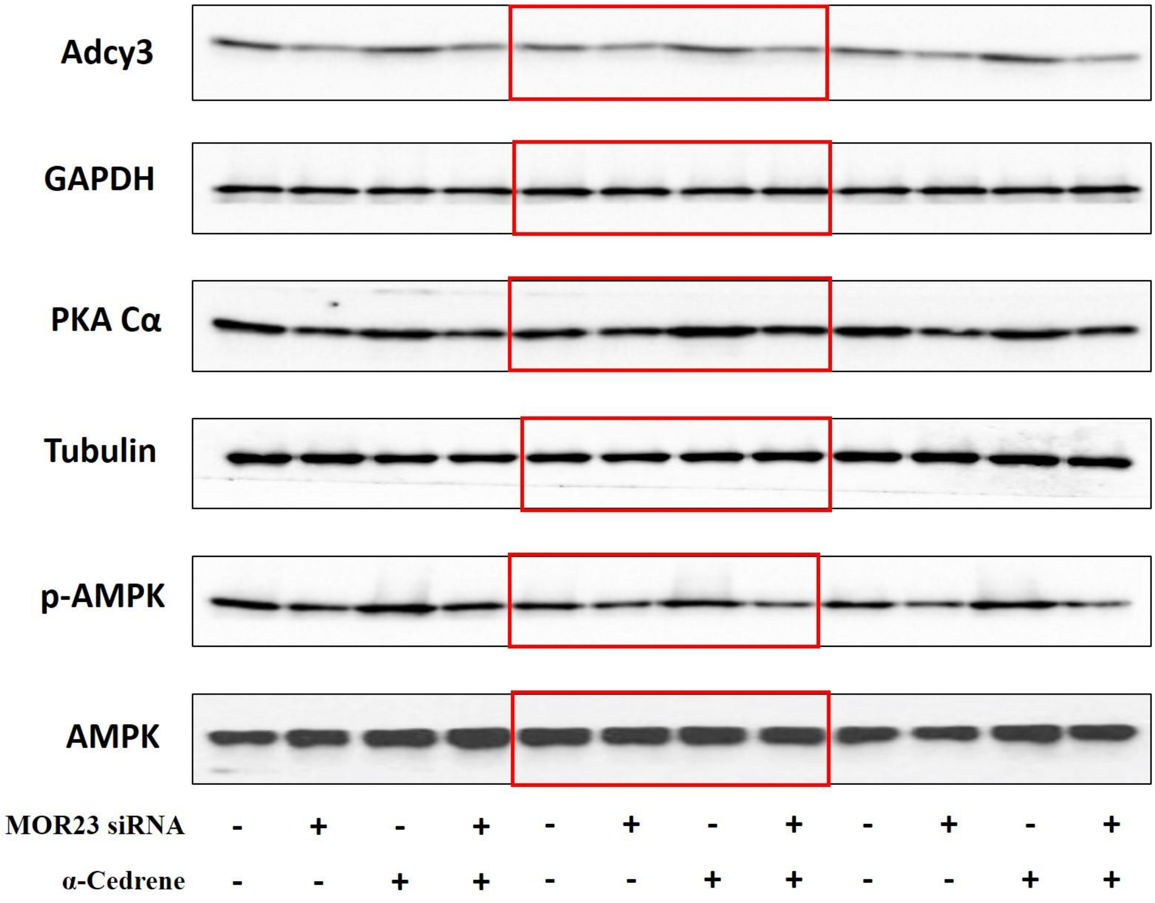

Supplementary Figure 2. Full length western blot membranes relative to Figure 2A. Red boxes indicate the cropping lines used to generate the figures.

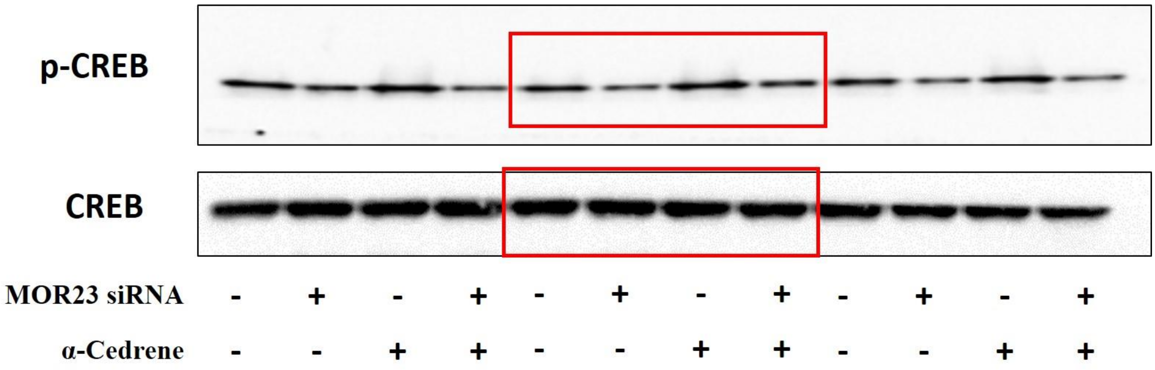

Supplementary Figure 3. Full length western blot membranes relative to Figure 4A. Red boxes indicate the cropping lines used to generate the figures.
